# Psychometric Properties of the Iowa Fluoride Study Oral Health Questionnaire in Mexican Adolescents

**DOI:** 10.7759/cureus.51165

**Published:** 2023-12-27

**Authors:** Gabriel Canseco-Prado, Blanca S González-López, Norma L Robles-Bermeo, María de L Márquez-Corona, Mirna I Minaya-Sánchez, Josué Bermeo-Escalona, Chrisel Zárate-Díaz, Adriana A Cabrera-Ortega, Carlo E Medina-Solís, Gerardo Maupomé

**Affiliations:** 1 School of Behavioral Sciences, Autonomous University of the State of Mexico, Toluca, MEX; 2 Academic Area of Dentistry of Health Sciences Institute, Autonomous University of Hidalgo State, Pachuca, MEX; 3 Advanced Studies and Research Center in Dentistry, "Dr. Keisaburo Miyata" of School of Dentistry, Autonomous University of State of Mexico, Toluca, MEX; 4 School of Dentistry, Autonomous University of Campeche, Campeche, MEX; 5 Department of Epidemiology, Richard M. Fairbanks School of Public Health, Indiana University-Purdue University, Indianapolis, USA

**Keywords:** adolescents, oral health, quality of life, cultural adaptation, validation

## Abstract

Background and objective: The perception of quality of life about health status is subjective; assessment of the impact includes well-being while eating, speaking, smiling, interacting with others, and feeling good about the esthetic appearance of teeth and mouth. The objective of the present study was to cross-culturally adapt and determine the validity and reliability of the Mexican version of the Iowa Fluoride Study Oral Health for adolescents.

Material and methods: A cross-sectional study was carried out in a sample of 240 Mexican adolescents aged 15 to 19 years. The questionnaire was translated, back-translated, and administered through the Google Forms platform. The cultural adaptation consisted of the evaluation of the grammatical, conceptual, and linguistic equivalences. The evaluation of the utility and acceptability was carried out through an analysis of semantic equivalence. The utility of the questionnaire was also evaluated by analyzing its grammatical readability. Reliability tests, Kaiser-Meyer-Olkin (KMO), factor analysis, and Pearson's correlation were performed.

Results: The mean age of all participants was 16.4±1.4; 65.3% (n=158) were female. Face validity was considered adequate. The wording of the objective and instructions of the questionnaire were improved. The confidentiality assurances were highlighted. The questions were clear, understandable, and pertinent, and they showed adequate syntax. The INFLESZ index corresponds to a "fairly easy" level of readability. In the quantitative validation, the correlation of items was greater than 0.4. The KMO was 0.930 (p=0.001), and Bartlett sphericity was 2466.5 (p=0.001). Through the exploratory factorial analysis, we evaluated the emotional well-being (12 items), social welfare (five items), and oral symptoms (OS) (three items) dimensions. Internal consistency was high (Cronbach’s α=0.942).

Conclusion: The culturally translated and adapted questionnaire is valid and reliable for use in research on Mexican adolescents.

## Introduction

Many factors can influence caries [1-3] and periodontal disease [3,4] risks in adolescents at the individual, family, and community level. In addition, poor oral hygiene, limited exposure to fluoride toothpaste, and a carbohydrate-rich diet contribute to these problems [5]. The oral health status (including adolescent behavior along psychological and sociocultural dimensions, as well as the socioeconomic environment where the individual lives) may influence adolescents’ physical, social, and emotional well-being (EW) [6,7].

The perception of quality of life in relation to health status is subjective; assessment of the impact includes well-being while eating, speaking, smiling, interacting with others, and feeling good about the esthetic appearance of teeth and mouth [8,9]. Questionnaires to assess the impact on quality of life have been created for adolescents, encompassing age, language, and sociocultural aspects pertinent to the region and/or country where they live [6,7]. Their validation and cultural adaptation have allowed their use in different populations, such as in Mexico, where previous studies have ascertained the impact of caries, fluorosis and malocclusion on the quality of life in adolescents, including socio-emotional implications [10-12]. Some of the questionnaires that measure the effect of oral health on the quality of life in adolescents incorporate the severity of the oral conditions and the extent of damage [13,14]. The Child Perception Questionnaire (CPQ 8-10) and CPQ 11-14 were designed for such age groups; they encompass bio-psycho-social aspects of oral health and their relationship with everyday life [15,16]. Specifically, the CPQ 11-14 assesses the impacts of oral health on quality of life in the preceding three weeks. It is made up of 37 items in four dimensions: oral symptoms (OS) (six items), functional limitations (nine items), emotional well-being (nine items), and social well-being (SW) (13 items). The CPQ 8-10 assesses impacts in the preceding four weeks-also in the same four dimensions but in 25 items: oral symptoms (five items), functional limitations (five items), emotional well-being (five items), and social well-being (10 items) [17]. They have been validated and culturally adapted in various countries and languages. Using Likert-type scales, items are scored as “Never”=0; “1-2 times”=1; “Sometimes”=2; “Often”=3; “Every day/Almost every day”=4. The CPQ encompasses the scores in each one of the dimensions; the range for CPQ 8-10 is 0-100, and the range for CPQ 11-14 is 0-148. Higher scores indicate a worse quality of life [18]. The advantages of the CPQ include their efficacy and ease of administration [19], with some limitations ascribable to age ranges [20]. They have been used in Mexican adolescents 11 to 14 years [21]. The instruments have become standard tools in the clinical and research armamentarium.

A variant of the CPQ 11-14 [22] commonly used is the Oral Health Questionnaire derived from the Iowa Fluoride Study [23]; it is a multidimensional questionnaire used in a prospective cohort evaluation to estimate the impact of caries, periodontal disease, dental fluorosis, malocclusions, and gingival bleeding on the perception of quality of life among children, adolescents, and their parents. It encompasses 48 questions, including 11 questions about the adolescents' opinion on the appearance of dental, bone, and soft tissue structures in the mouth. There were changes in the naming of domains: oral problems (OP) instead of oral symptoms (OS), which also integrated the dimension functional limitation (FL); the domain emotional well-being (EW), about feelings; and social well-being (SW), called leisure time activities [23].

The objective of the present study was to cross-culturally adapt and determine the validity and reliability of the Mexican version of the Iowa Fluoride Study of Oral Health in adolescents.

## Materials and methods

Design, population, and sample

A cross-sectional study was conducted on a simple random sample of adolescents ages 15 to 19 years. They were registered as post-elementary students in a public school located in an urban area of Pachuca de Soto, México. It is the capital city of the state of Hidalgo in Mexico. Census data from 2020 indicated a total population of 297,848 inhabitants. In 2015, Pachuca had a Human Development Index of 0.834 (considered very high).

Taking into account the number of items on the scale, five adolescents were added for each item [24]. The selection criteria were high school adolescents, either sex, from the city of Pachuca, who had given informed consent. For those under 18 years of age, parents gave informed consent for their participation, and adolescents assented. Participants had to be able to read and write Spanish. Exclusion criteria were adolescents with systemic diseases and/or with a physical disability hindering adequate oral self-care. Questionnaires with more than five unanswered items were excluded. The questionnaire was administered to a total of 306 adolescents; 66 questionnaires had no record of age or had an age different from that established in the selection criteria. They were excluded. The analysis was performed on 240 questionnaires.

Translation

A pre-established protocol was used for cross-cultural adaptation [25]. The strategy for translation comprised the following steps: (a) assessment of clarity (use of simple words), (b) adherence to colloquial language (avoid dental lingo), and (c) analysis of semantic equivalence (representing original content). Investigators who had the following qualities conducted the translation: appropriate English proficiency and proven experience translating questionnaires for dental research. We started with the first translation by a bilingual dental researcher; another two researchers reviewed separately the translated questionnaire and recorded their agreements and disagreements with the first translation. There was generally very good agreement. Translators subsequently met as a panel to discuss discrepancies and reached a consensus on a second version. The back-translation was separately undertaken by a public health dentist with substantive expertise in social and behavioral sciences, as well as fluent in both English and Spanish. The panel of translators then discussed this back-translation, examining the third version's coherence and understandability in the context of the original questionnaire. The final (fourth version) version was pilot-tested with a sample of 15-19-year-old teenagers (not included in the main study). Their observations were considered and incorporated after evaluation by the panel.

Cultural adaptation

Three academics and ten adolescents of similar age as the target population evaluated the translated questionnaire for face validity. First, a focus group of dentists evaluated the grammatical, conceptual, and linguistic equivalences. To appraise the utility and acceptability of the questionnaire, the adolescents assessed the semantic equivalence, comprehension, clarity, readability, wording, and time to respond to the questionnaire [26]. Subsequently, a pilot test was conducted on a sample of 15-19-year-old teenagers (not included in the main study).

Data collection

The questionnaire was adapted for completion in the Google Forms platform (due to the COVID-19 pandemic, it could not be administered face-to-face to participants). Information about the study was included in an invitation to the adolescents and/or parents/guardians through electronic media. The contact information was supplied by the school under the provision of confidentiality and authorizations by adolescents and/or parents/guardians.

Statistical analysis

To estimate the reliability of the questionnaire, we assessed internal consistency through Cronbach’s alpha, yielding an acceptability value ≥0.80. The Kaiser-Meyer-Olkin test (KMO≥0.8) was used to evaluate the sample adequacy and the suitability of the use of factor analysis with Bartlett's test p≤0.05. Exploratory factor analysis (EFA) was used to analyze the dimensions, the relationship of the items, and the corresponding construct, using VARIMAX rotation analysis of the components.

The evaluation of correlation across items was carried out with Pearson's correlation analysis; significant positive correlations were accepted, with a correlation coefficient ≥0.4. Statistical analysis was performed with SPSS Software Version 20.0 (IBM Corp., Armonk, New York, USA).

Evaluation of utility

The utility was evaluated considering administration time, the simplicity of the format, wording, and comprehension of the questions by means of grammatical readability analysis [27].

Ethical considerations

The protocol was approved by the ethics committee of the Autonomous University of the State of Mexico (registration number 2021 P02). The study adhered to the ethical standards of the Declaration of Helsinki and the federal guidelines for research on human subjects, NOM-012-SSA3-2012, in the Mexican Regulations of the General Health Law on Health Research.

The adolescents who participated in the study gave their consent; for minors, their parents or guardians granted their informed consent after being fully informed of the nature of the study and the data privacy notice. The informed consent form and assent verification were secured through a secure internet platform and electronically signed by participants or their parents or guardians.

## Results

The study included 240 adolescents ages 15 to 19 years, with a mean age of 16.4±1.4; 65.3% (n=158).

Validity

The face validity was estimated through two groups; one had four teenagers, and the second had four dental researchers. Face validity was established through an instrument to evaluate clarity, precision, and understandability at the individual level, using a scale of three levels: 1=criterion absent, 2=appropriate criterion, and 3=highly apparent criterion. There were entries for comments and observations.

We used the simple concordance index to quantify the degree of concordance among raters for clarity, precision, and understandability. Complete concordance was pi=1, generally considered adequate when >0.80 for each item.

The items rated according to the three categories reached levels of clarity, precision, and understandability thresholds >0.8. The participating researchers suggested emphasizing the purpose of the scale and the importance of confidentiality; moreover, they pointed out that only the minimum necessary instructions be used. They also requested that the wording of some concepts in the Likert scale prompts be improved. They commented that the questions met the criteria of clarity, relevance, adequate syntax, pertinence, and comprehensibility.

The participating adolescents addressed all questions, reported that the questions were clear and easy to understand, and had no negative comments about the length of the scale. In addition, they commented: "I was very interested since my body issues always come from my teeth," "These questions were interesting," and "The questions were very well posed." Face validity was considered adequate.

The INFLESZ [28] readability index of items was 71.11±24.12, corresponding to a "fairly easy" level. No misspelled words were identified, and only the words "Iowa" and "maxillae" were considered rare and/or difficult to understand.

Evaluation of utility

To evaluate the utility of the questionnaire, we considered whether the items were easy to read, if the time to administer the questionnaire was not burdensome (under twenty minutes), whether the form was easy to fill out, and if the wording and understandability of the questions were appropriate. All these aspects were satisfactory.

Quantitative validity

Reliability

The total Cronbach's alpha for the final set of items was 0.942, suggesting excellent reliability. The reliability assessment of the dimensions of emotional well-being (EW), social well-being (SW), and oral symptoms (OS) yielded satisfactory values (Table 1).

**Table 1:** Evaluation of reliability of items across dimensions and of the total set of items using Cronbach's alpha coefficient.

Component	Number of items	Reliability test
Emotional well-being	12	0.950
Social well-being	5	0.793
Oral symptoms	3	0.797
Total items	20	0.942

Exploratory factor analysis

The selected items showed a correlation equal to or greater than 0.4. Only 19 items were eliminated from the 48 that originally made up the questionnaire.

The Kaiser-Meyer-Olkin (KMO) sample adequacy measure was 0.930 (p=0.001), and the items (criterion validity) with a correlation coefficient ≥0.4 were selected. Bartlett's test of sphericity was 2466.5 (p=0.001), demonstrating independence between variables and suggesting that the use of factor analysis was appropriate.

**Figure 1:**
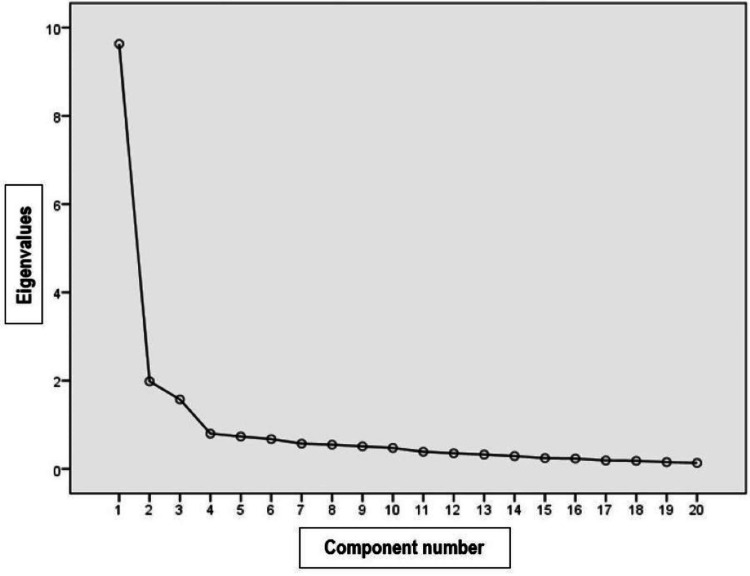
Scree plot (selection of factors). Note: Scree plot is a line plot of the eigenvalues of factors or principal components in an analysis. The scree plot is used to determine the number of factors to retain in an exploratory factor analysis (FA) or principal components to keep in a principal component analysis (PCA).

Twenty components were obtained using the principal component extraction method, the first three of which presented values >1.0 and were selected (Table 2). It was observed that the most important factors were found in the highest part of the scree plot (Figure 1).

The first component explained the highest percentage of total variance, indicating that there was a relationship between the variables of the EW dimension and the quality-of-life scores. The second component SW and the third component OS showed an association between five and three of the variable quality of life scores. The three components explained 65.9% of the variance, supporting a theoretical relationship between the items.

**Figure 2:**
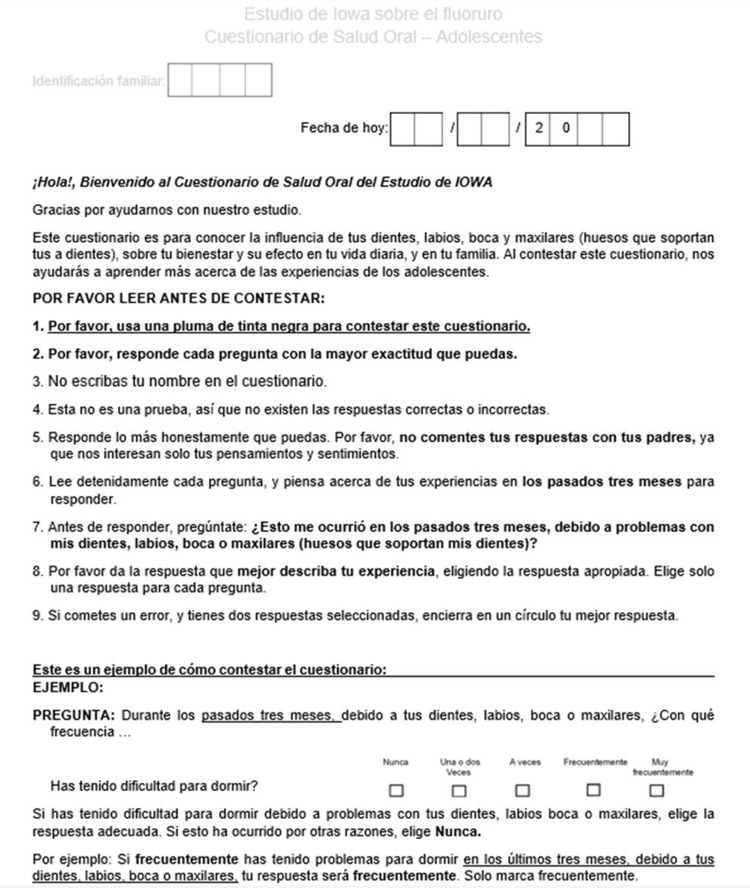
Appendix, Questionnaire.

**Figure 3:**
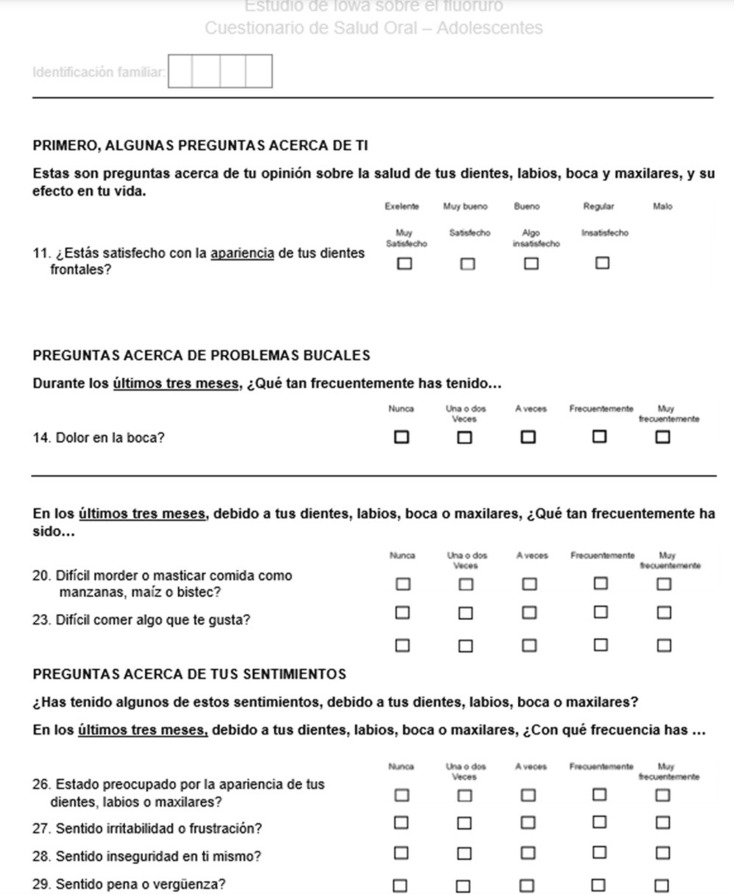
Appendix, Questionnaire.

**Figure 4:**
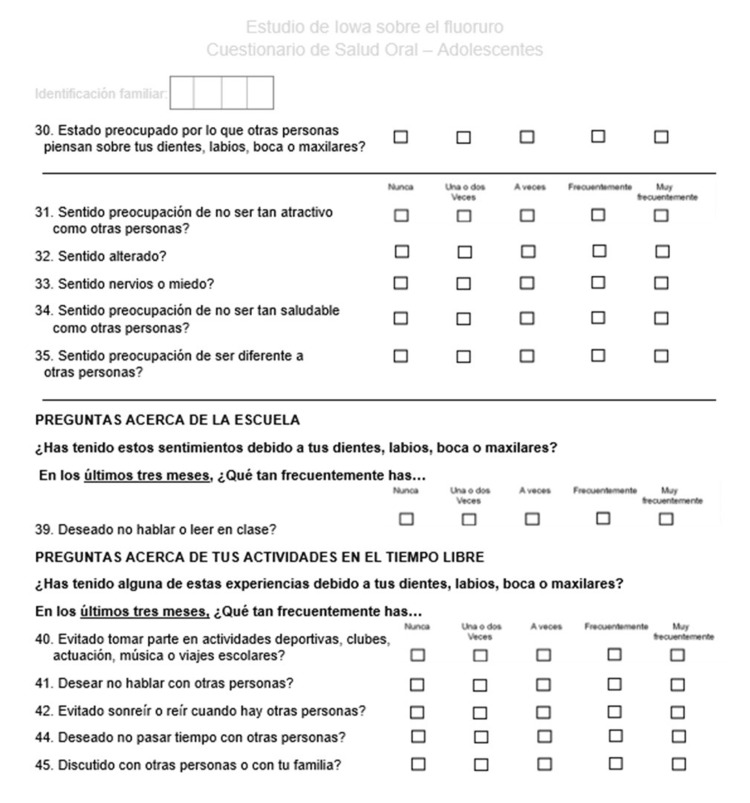
Appendix, Questionnaire.

The final version of the questionnaire had 20 items, after excluding 28 out of a total of 48 original items (Appendix 1, Figures 2, 3, 4).

**Table 2:** Components obtained through the principal component analysis extraction method (the first three were selected). EW: emotional well-being, SW: social well-being, OS: oral symptoms.

Item number	The name assigned to the item	Components		
		EW	SW	OS
30	Worried about what people think of your mouth	0.859		
31	Worried about being unattractive	0.838		
28	Felt insecure	0.812		
29	Felt embarrassed	0.782		
35	Worried about being different	0.728		
42	Avoided smiling	0.725		
32	Felt upset	0.710		
34	Worried about being unhealthy	0.693		
26	Worried about your oral appearance	0.691		
33	Felt nervous	0.625		
27	Felt irritable	0.599		
11	Satisfied with your dental appearance	0.545		
41	Wished not to talk		0.743	
44	Not spending time with people		0.707	
39	Wished not to talk or read		0.687	
45	Argued with someone		0.630	
40	Avoided extracurricular activities		0.610	
23	Difficulty eating what he/she likes			0.823
20	Difficulty biting			0.783
14	Mouth pain			0.709

## Discussion

The objective of the present study was to cross-culturally adapt and determine the validity and reliability of the Mexican version of the Iowa Fluoride Study Oral Health Questionnaire for adolescents. The original CPQ11-14 may encompass a slightly different domain naming in the questionnaire; we occasionally refer to CPQ11-14 components when they are more amenable to comparing the results of similar studies in Mexico and in Spanish-speaking countries [29]. The CPQ11-14 is a multidimensional questionnaire including 37 questions in four domains: OS, FL, EW, and SW [30]. It has been translated into several languages, validated, and administered in different sociocultural contexts for clinical and epidemiological studies [31-33]. The CPQ 11-14 was translated into Spanish and culturally adapted for application in Chilean adolescents; the cultural context of Chile is likely somewhat different from Mexico’s [34]. It has been used in Mexico to assess the impact of caries and fluorosis in adolescents aged 11 to 12 years [21]. A questionnaire may have good or acceptable reliability in a sample of subjects but not necessarily in another population group or a different region, even in the same country; it is, therefore, desirable to consider the properties [35] and the differential performance of each item. Differences have been observed in responses to items in the FL, SW, and EW dimensions by ethnicity [36].

There are few reports about the perceived impact of health status on quality of life in adolescents over 14 years of age using the CPQ 11-14 [37]. It has been suggested that the CPQ 11-14 should not be administered to adolescents outside the age range originally targeted because there are emotional maturity differences in the perception of the relationship between health status and quality of life. At 15 to 17 years of age, decision-making is largely governed by impulses, while at the same time, they have already increased their capacity for abstract thinking, comparing themselves with their peers, developing interests in different areas, and establishing future goals. These characteristics may be leveraged to inform the design of oral health promotion programs [38].

Through the exploratory factorial analyses, we were able to appraise the EW, SW, and OS. These can be compared to the two dimensions other authors have preferred, supporting the observation that sub-scales with scarce items may increase the likelihood of error [19]. The final version we assembled had 20 items: 12 in EW, five in SW, and three in OS. Statistical assessments were appropriate. The items “Worried about what people may think of your mouth,” “Worried that I may not be attractive to others,” and “I felt insecure” had the largest factorial load in the 12 items in EW; this may be explained by this age group feeling emotionally vulnerable, with physical appearance being crucial. Oral health status may be assumed to have an impact on emotional and social features, perhaps enhancing the importance of dental care to support quality of life. Scores for SW items such as “I did not want to talk,” “Did not want to spend time with peers,” and “I did not want to speak or read aloud” reinforced the perception that their appearance makes them feel insecure. As far as OS items are concerned, only “I find it difficult to eat preferred foods,” “Biting is difficult,” and “My mouth hurts” were associated with an impact on daily life.

The Cronbach’s alpha coefficient we obtained was 0.942, suggesting that the instrument maintained its psychometric properties when compared to other reports gauging validity and reliability upon cross-cultural adaptation, e.g., in Shiraz, Iran (0.855) [19], in Southern Chile (0.85) [29], and in Northern India (0.963) [16]. For our SW (0.793) and OS sub-scales (0.797), their internal consistency was adequate even with fewer items, thereby suggesting we could evaluate the constructs even with our shorter questionnaire. 

The criterion validity of items in the scale was considered acceptable, further supporting the stability of the questionnaire [39]. We eliminated items with a value lower than 0.40 in the Pearson correlation as well as those that in the first-factor analysis showed no relationship with any of the components and that obtained values below 0.50 in the rotated component analysis. The lowest factorial load was 0.308 in the case of the item addressing teeth color, and the highest was for the item worried about what people think of your mouth, with a 0.859 value. We eliminated items with factorial loads below 0.5 since they could be expected to have little influence on the validity of the questionnaire.

The INFLESZ readability index corresponded to "fairly easy" reading. It was decided to eliminate the word Iowa because it is the unfamiliar name of a state in the United States, and the word maxillae was changed to bones that support the teeth; the original word was difficult to understand. The final version of the questionnaire showed semantic equivalence, idiomatic equivalence, and the correct meaning of words and grammar, according to the analysis of the expert test for cultural validation.

The present study had some limitations that must be considered to adequately interpret the results. Due to the COVID-19 pandemic health emergency, it was unfeasible to perform a clinical examination to diagnose oral diseases and conditions and link them to the impact on the perception of quality of life. The final questionnaire should ideally be administered through face-to-face interviews; such contact ought to gather, at a minimum, DMF scores and a periodontal/gingival index. Our findings may be applied to future studies on Mexican adolescents, possibly adding aspects that influence the perception of oral health status, e.g., socioemotional, sociocultural, socioeconomic, and sociodemographic aspects, as well as dental care utilization and patterns of food consumption.

## Conclusions

The psychometric properties of the adapted questionnaire are appropriate for use in Mexican adolescents. Its utilization in similar environments is feasible (with appropriate reservations), accruing important information about individual impacts derived from dental conditions. The validated instrument could be a powerful tool to expand the quality of life data in Mexico; it offers a developmental path for subsequent revisions relevant to other regions and countries in Latin America in general. Its main strength resides in the multi-faceted exploration of real-life issues in the context of oral health: pain, discomfort, function and social appearance, to name a few. Allowing dental professionals to objectively gauge impacts in environments with unequal access to dental care or affected by the unequal distribution of disease is an important asset to designing population-relevant health programs. Moreover, a standardized tool opens the opportunity to make reasonable contrasts across settings, e.g., in multiple cities or across countries, while controlling for influencing/system factors. Considering its already widespread utilization in multiple countries, an adapted tool supports applications in the clinical, policy, program planning/evaluation, and research environments.

## Appendices

Appendix 1
